# A randomized trial to evaluate a launderable bed protection system for hospital beds

**DOI:** 10.1186/2047-2994-1-27

**Published:** 2012-07-26

**Authors:** Edmond A Hooker, Steven Allen, Larry Gray, Cynthia Kaufman

**Affiliations:** 1 Department of Health Services Administration, Xavier University, 3800 Victory Parkway, ML 5141, Cincinnati, OH 45207-7331, USA; 2 Department of Pathology and Laboratory Medicine, Indiana University School of Medicine, IU Health Pathology Laboratory, 350 W. 11th Street, Indianapolis, IN 46202, USA; 3 TriHealth Clinical Microbiology Laboratory, 619 Oak Street, Cincinnati, OH 45206, USA; 4 IU Health Pathology Laboratory, 350 W. 11th Street, Indianapolis, IN 46202, USA

## Abstract

**Background:**

Hospital beds are potential reservoirs of bacteria in hospitals. Preventing contamination of the bed and providing a cleaner surface should help prevent hospital-acquired infections (HAIs). Most hospital beds are cleaned between patients (terminal cleaning) using quaternary ammonia compounds (quats).

**Objective:**

The study had two objectives: identify levels of bacterial contamination on beds (including the mattress and bed deck) and evaluate a new launderable cover.

**Methods:**

Hospital beds on a bariatric surgery ward were randomized to either receive or not receive a launderable cover (Trinity Guardion, Batesville, IN). Bacterial counts on the surface of the mattress, the bed deck, and the launderable cover were then collected using Petrifilm™ Aerobic Count Plates (Petrifilm™, 3M™, St. Paul, MN, USA) (Petrifilm™) at three time periods (before patient use, after discharge, and after terminal cleaning). Standard hospital linen was used in all rooms.

**Results:**

The launderable cover (n = 28) was significantly cleaner prior to patient use than were the cleaned mattresses (n = 38) (1.1 CFU/30 cm^2^ vs. 7.7 CFU/30 cm^2^; p = 0.0189). The mattresses without launderable covers became significantly contaminated during use (7.7 CFU/30 cm^2^ on admission vs. 79.1 CFU/30 cm^2^ after discharge; p < 0.001). The mattresses with launderable covers did not become contaminated (3.0 CFU/30 cm^2^ on admission vs. 2.5 CFU/30 cm^2^ at discharge; p = 0.703). After terminal cleaning, the mattress surface contamination decreased to 12.8 CFU/30 cm^2^ (median 3 CFU/30 cm^2^; SD 7.8), but the bed deck was more contaminated (6.7 CFU/30 cm^2^ after discharge compared to 30.9 CFU/30 cm^2^ after terminal cleaning; p = 0.031).

**Conclusions:**

Terminal cleaning fails to eliminate bacteria from the surface of the hospital mattress. The launderable cover provides a cleaner surface than does terminal cleaning with quats, and the cover protects the bed from contamination during use.

## Introduction

It has been long recognized in the surgical arena that sterile conditions are important in order to prevent wound infections. Until recently, less emphasis has been placed on the environmental contamination outside of the operating room. In the United States, it has been estimated that hospital-acquired infections (HAIs) kill over 100,000 people each year and cost in excess of 35 billion dollars [1-3].This is mortality equivalent as a 300-passenger airliner fatally crashing each day.

It is imperative that all sources of HAIs be identified, addressed and cleaned as well as possible. In every hospital room, there is a patient bed, which is comprised of a bed deck (frame) and a mattress. The mattress consists of different inner workings (e.g., foam or more sophisticated air mattresses) and a permanent cover that is not removed between patients. These permanent covers are most commonly made of polyurethane, nylon, or Gore-tex**®** surface. These mattresses must be cleaned between patients, along with the other parts of the patient room. The Centers for Disease Control and Prevention (CDC) recommended in 2003 mattresses be cared for in such a manner to prevent the spread of HAIs. In the 2003 publication on environmental infection control in healthcare facilities, the CDC acknowledged that hospital mattresses can become contaminated during patient care [4]. They recognized that the linen sheet is not a cover and that mattresses should be replaced when torn, punctured or stained. They recommended that mattresses be cleaned and disinfected between patients using Environmental Protection Agency (EPA)- registered disinfectants that are compatible with the mattress cover materials to prevent tears, cracks and holes in the cover. Finally, the CDC recommend laundering mattress covers that are made of fabric and all pillow covers.

The Centers for Disease Control and Prevention (CDC), in its most recent guideline for disinfection and sterilization in healthcare facilities, considers many areas of the patient room to be “non-critical” and does not make specific recommendations for cleaning standards for these areas [5]. According to this guideline, bedrails are non-critical surfaces. The mattress and bed deck are not specifically adressed in the new guidelines. Several studies have shown that the entire hospital room, and especially the hospital bed, commonly remains contaminated even after terminal cleaning, the cleaning that occurs after the patient is discharged [6-9]. Recent studies have shown that, if the previous occupant had infections with methicillin-resistant *Staphylococcus aureus* (MRSA), vancomycin-resistant *Enterococcus* (VRE), or *Clostridium difficile*, there is a significantly increased risk of the subsequent patient contracting diseases caused by these organisms [10-12].

Several studies have documented the hospital mattress as a vector for HAIs. In England, two different research teams have isolated *Pseudomonas aeruginosa* inside mattresses during outbreaks of *P. aeruginosa* at their facilities [13,14]. Another English hospital cultured MRSA from foam mattresses during an outbreak of MRSA [15]. In the United States, Canada, and England, antibiotic-resistant strains of *Acinetobacter* have been isolated from mattresses during outbreaks of *Acinetobacter* in burn units [16-18]. One incubator mattress was implicated in an outbreak of neonatal gastroenteritis with *Samonellawien*[19]. There is published evidence that demonstrates enhanced cleaning of the hospital room may help decrease the transmission of infections from the environment [20-23].

In the hospital environment, other than the operating room, the guiding principle for cleaning has been to decrease contamination without any attempt to achieve sterilization. Three authors have proposed levels of bacterial contamination that might be an acceptable standard for assessing terminal cleaning of hospital rooms [24-26]. Unfortunately, there is no evidence that these levels of contamination are low enough to help prevent HAIs.

In order to provide the cleanest possible environment for patients, many hospitals have enhanced cleaning efforts in rooms. Curtains are no longer simply cleaned when they are visually soiled; they are replaced after each patient use because research has shown how contaminated curtains can become. Some hospitals are testing the use of ultraviolet lights and hydrogen peroxide as means to more effectively kill bacteria and decrease hospital-acquired infections. However, one area that has not received enough attention is the hospital bed. Of all of the areas in the hospital room, the hospital bed is the object with which patients have the most contact.

Determining the level of bacterial contamination in hospital rooms is critical to developing housekeeping methods to decrease HAIs. The Centers for Medicare and Medicaid Services have recently mandated that hospitals enact an infection prevention and control program that includes “appropriate monitoring of housekeeping…to ensure that the hospital maintains a sanitary environment.” [27]

In 2011, Trinity Guardion (Batesville, IN) developed a launderable cover for hospital beds. This cover is engineered to protect the mattress and the bed deck from contamination, and it allows hospitals to launder the cover between patients. There are no published studies evaluating the performance of these covers in the clinical environment.

The current research focuses on the potential ability of these new launderable covers to reduce bacterial contamination of hospital beds. Prior research on bacterial contamination in hospitals has used a number of methods for enumerating bacteria on surfaces. A commonly reported method is to utilize moistened cotton-tip or rayon-tipped swabs [7,12,28,29]. In recent years several authors have used contact plates, Replicate Organism Detection and Counting (RODAC™) and contact slides (DIPSLIDE™), to detect and enumerate bacteria on environmental surfaces [8,30]. Other authors have described using gauze to rub the surface [18].

In the food industry, it is common to use Petrifilm™ plates (3M™, St. Paul, MN, USA) to enumerate bacterial counts in food processing plants. These plates are easy to use and do not require the use of a microbiological laboratory. One researcher used the Petrifilm™ Staph Express Plates to detect methicillin-resistant *Staphylococcus aureus* (MRSA) on egg-crate mattresses [31].

The current research had three goals. The first goal was to evaluate how well terminal cleaning eliminates bacteria from hospital beds (mattresses and bed deck). The second goal was to compare bacterial contamination of beds after terminal cleaning to the contamination on a laundered cover. The third goal was to compare contamination on beds protected by a sheet to those covered with a launderable cover and a sheet.

## Materials and methods

### Petrifilm™

Several styles of Petrifilm™ plates are available to identify bacterial contamination on surfaces. Petrifilm™ Aerobic Count Plates (Petrifilm™) consist of standard bacterial nutrients in a cold-water soluble gel. The gel is manufactured and applied as a thin coating onto a sheet of clear flexible plastic material measuring 75 mm by 95 mm. The plates are dehydrated and packaged 50-plates-per-pack. Plates must be hydrated with buffered peptone water broth prior to use. Red colonies on the gel identify bacterial growth. Hydrated plates were stored in clean plastic bags at 4 °C for up to two weeks prior to use. Each hydrated plate was applied to the surface being sampled for 10 seconds. Sampling was standardized so that samples were all taken from the center of the top of the mattress and bed deck.

### Hospital mattress

All beds used in the study were Hill-Rom VersaCare**®** beds. The beds’ mattresses each had an air mattress core and a cover made of non-permeable stamped layers of polyurethane coated nylon. All beds were located on a bariatric surgery unit of a community hospital.

### Launderable mattress cover

**Figure 1:**
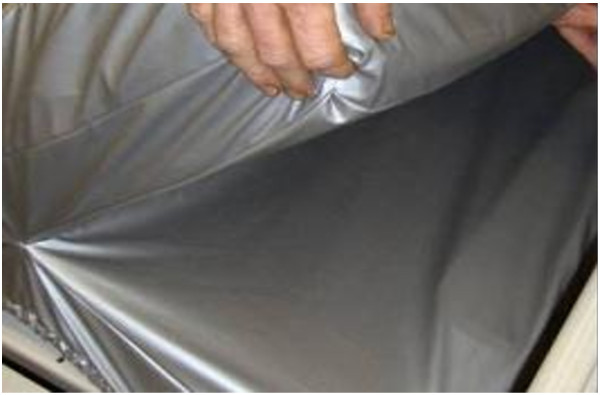
Launderable cover by Trinity Guardion showing how the cover protects both the mattress and bed deck.

The cover is made of a fluid-impermeable, breathable polyurethane fabric with knitted polyester backing. It is manufactured using welded seams (Trinity Guardion, Batesville, IN, USA) (Figure 1). Pockets on each end encapsulate the mattress and the cover fits snugly around the bed deck to keep fluids from being trapped between the bed deck and the mattress. The cover does not interfere with the operation of the bed and can be laundered in hot or cold water. Chlorine use does not damage the cover. During the study, each cover was laundered between uses in a commercial laundry using multiple cycles with detergent, chlorine bleach, and multiple hot water rinses. Covers were then dried at over 200**°**Fahrenheit (93**°**Celcius) for 30 minutes.

### Bed deck

The bed deck is the metal frame upon which the hospital mattress rests.

### Randomization

Beds were randomized to receive or not receive a launderable mattress and bed deck cover (launderable cover). Randomization was performed using a chart that assigned beds based on the time the page for terminal room clean request was received. Upon receiving a page that the room was ready to be cleaned, nursing staff determined if the bed would receive a launderable cover or not. Environmental services personnel cleaned the rooms, and were aware of the ongoing study of cleanliness of the mattresses.

### Cleaning of the standard mattress and bed deck

All beds were cleaned using EnverrosSanimaster 4 (Ecolab Inc., St. Paul, MN) diluted 1:128. The disinfectant was prepared using an automated mixing system, and clean rags were used once and never returned to the cleaning solution. Beds were not cleaned using detergent prior to disinfection and were not rinsed after disinfection.

### Sampling procedure

After rooms were terminally cleaned, the mattress surface and the bed deck of all beds were sampled. If the bed received a cover, the cover was sampled after being applied to the bed. All mattresses, with or without a launderable cover, were covered with clean sheets. When the patient was discharged, the bed linen was removed and the mattress surface and bed deck of all beds were sampled prior to terminal cleaning. If the mattress had received a launderable cover, that cover was sampled and then removed. The underlying mattress surface was then sampled. Environmental services then cleaned the bed, and the mattress surface and bed deck were again sampled. If the patient was discharged in less than 24 hours, the bed was removed from the study.

### Laboratory procedure

Petrifilm™ plates were incubated for 24 hours at 37 °C in 5-7 % CO2. After incubation, plate counts were performed visually. A red colony within the hydrated area was counted as one colony-forming unit (CFU). All counts were per 30 cm^2^. Counts of greater than 300 CFU per 30 cm^2^ were recorded as “too-numerous-to-count” (TNTC). After performing the count, the Petrifilm™ plate was blotted onto 5 % sheep blood agar medium (BA). Growth was examined after BA was incubated overnight at 37 °C in 5-7 % CO2. Bacteria were identified using standard laboratory methods.

### Statistics

All calculations were performed using IBM™ SPSS™ for Macintosh, version 19 (International Business Machines Corporation, Armonk, NY, USA). Growth was quantified using descriptive statistics. The t-test compared the means.

## Results

Sixty-six beds were entered into the study: 38 that did not receive a launderable cover and 28 that did receive a launderable cover. All beds had been terminally cleaned using quaternary ammonia compound prior to being placed in the study, and after use, each bed was again cleaned using quaternary ammonia compound. The average length of stay was similar for both groups (89 hours for beds without a cover and 77 hours for those with a cover; p = 0.530).

The launderable cover was significantly cleaner prior to patient use than were the mattresses, which had been terminally cleaned (p = 0.0189) (Table 1). The mean colony count, prior to patient use, on the 38 mattresses that did not receive a launderable cover was 7.7 CFU/30 cm^2^ (median 3 CFU/30 cm^2^ ; SD 14.5). The mean colony count, prior to patient use, on the launderable cover was 1.1 CFU/30 cm^2^ (median 1 CFU/30 cm^2^; SD 1.2).

**Table 1:** Launderable Cover provides a cleaner surface and prevents mattress contamination better than terminal cleaning with quaternary ammonia compound

	**Before Patient Use**^1^**(CFU/30 cm**^**2**^**)**	**After Discharge**^**2**^**(CFU/30 cm**^**2**^**)**	**After terminal cleaning (CFU/30 cm**^**2**^**)**
Mattress Surface when NO Cover was used (n = 38)	7.7 ± 14.5 median = 3	79.1 ± 113.4 median = 25	12.8 ± 48.2 median = 3
Surface of Launderable Mattress Cover (n = 28)	1.1 ± 1.2 median = 1	108 ± 126 median = 35	1.1 ± 1.2^*^ median = 1
Mattress Surface when a Launderable cover was used (n = 28)	2.5 ± 2.7 median = 2	3.0 ± 5.9 median = 1	3.7 ± 8.0 median = 1

1. Before patient use all beds were terminally cleaned using quaternary ammonia compound. Launderable covers were laundered prior sampling. All beds were covered with clean sheets prior to patient use.

2. After discharge, but prior to terminal cleaning.

*After laundering.

After the patient was discharged, surfaces were again cultured. The mattresses that did not utilize a launderable cover had significant increases in their bacterial counts (7.7 CFU/30 cm^2^ (median 3 CFU/30 cm^2^; SD 14.5) on admission vs. 79.1 CFU/30 cm^2^ (median 25 CFU/30 cm^2^ ; SD 113.4) at discharge; p = 0.001). When the launderable cover was utilized, there was no significant change in bacterial counts on the surface of the mattress (2.5 CFU/30 cm^2^ (median 2 CFU/30 cm2; SD 2.7) on admission vs. 3.0 CFU/30 cm^2^ (median 1 CFU/30 cm2; SD 5.9) at discharge; p = 0.703).

Final testing was performed after the beds were terminally cleaned prior to the next patient. After this terminal cleaning, the mattress surface contamination was decreased from 79.1 CFU/30 cm^2^ (median 25 CFU/30 cm^2^; SD 113.4) to 12.8 (median 3 CFU/30 cm^2^; SD 48.2) in beds that did not use a cover, which is only an 84 % reduction (less than log 2 reduction).

### Contamination of the bed deck

The bed decks of all beds prior to patient use were more contaminated than the mattress surfaces. The mean colony count, prior to patient use, on the 38 bed decks of beds that did not receive a launderable cover was 33.0 CFU/30 cm^2^ (median 4 CFU/30 cm^2^ ; SD 100.8). The mean colony count, prior to patient use, on bed decks that received a launderable cover was 28.2 CFU/30 cm^2^ (median 2 CFU/30 cm^2^; SD 78.2) (Table 2).

**Table 2:** Launderable cover protects the bed deck from bacterial contamination during patient use

	**Before Patient Use**^**1**^**(CFU/30 cm**^**2**^**)**	**After Discharge**^**2**^**(CFU/30 cm**^**2**^**)**	**After terminal cleaning (CFU/30 cm**^**2**^**)**
Bed Deck when NO cover utilized (n = 38)	33.0 ± 100.8 median = 4	10.3 ± 25.9 median = 1.5	43.5 ± 101.6 median = 2
Bed Deck when Launderable Mattress Cover utilized (n = 28)	28.2 ± 78.2 median = 2	1.9 ± 3.6 median = 1	13.75 ± 56.4 median = 1
All Bed Decks (n = 66)	31.2 ± 91.2 median = 3	6.7 ± 20.2 median = 1	30.9 ± 86.1 median = 2

1. Before patient use all beds were terminally cleaned using quaternary ammonia compound.

2. After discharge, but prior to terminal cleaning.

After the patient was discharged, the bed deck was again tested. The bed deck that did not utilize a launderable cover had significantly more bacteria than did the bed decks that had utilized a cover (10.3 CFU/30 cm^2^ (median 1.5 CFU/30 cm^2^; SD 25.9) vs. 1.9 CFU/30 cm^2^ (median 1 CFU/30 cm^2^ ; SD 3.6); p = 0.011).

Final testing was performed on the bed deck after the beds were terminally cleaned. After this terminal cleaning, the bed deck was more contaminated than before terminal cleaning (Table 2) (p = 0.031). The mean colony count, after discharge, on the 66 bed decks was 6.7 CFU/30 cm^2^ (median 1 CFU/30 cm^2^ ; SD 20.2). The mean colony count, after terminal cleaning, on the 66 bed decks increased to 30.9 CFU/30 cm^2^ (median 2 CFU/30 cm^2^; SD 86.1). Most commonly the bacteria had been present on both the bed deck and mattress after discharge, but the counts were significantly higher after terminal cleaning. There was one bed where *P. aeruginosa* was present on the mattess after discharge but not on the bed deck. After terminal cleaning, the *P. aeruginosa* was present on both the mattress and the bed deck.

### Controls

The mean bacterial count for control plates (n = 62) was 0.05 CFU/cm^2^ (with only 3 plates having any growth).

### Bacteria identified

There were 6 samples taken for all beds in the study (one for the mattress surface and one for the bed deck at each of the three time points: pre-admission, dirty after discharge, and after terminal cleaning). An additional two samples were taken when a cover was utitilzed (one sample of the cover before use and one of the dirty cover after discharge). This gave a total of 452 samples. Many samples had multiple bacteria. The bacteria identified included coagulase-negative *Staphylococcus*, *Bacillus* species, *Micrococcus* species, *Pseudomonas fluorescens, Pseudomonas aeruginosa, Corynebacterium* species, mold, *Streptococcus*viridans*, Rhizobium radiobacter, Paracoccusyeei and Acinetobacter baumannii* (Table 3).

**Table 3:** Bacteria isolated from mattress, bed deck, and launderable cover

**Bacteria**	**Number of cultures with bacteria**	**Percent of total number of cultures**
Coagulase-negative *Staphylococcus*	281	62.2 %
*Bacillus* species	93	20.6 %
*Micrococcus* species	83	18.4 %
*Corynebacterium*	13	2.9 %
*Pseudomonas flourescens*	9	2.0 %
Mold	9	2.0 %
*Streptococcus viridians*	6	1.3 %
*Pseudomonas aeruginosa*	5	1.1 %
*Pseudomonas pseudoalcaligenes*	1	0.2 %
*Acinetobacter baumannii*	1	0.2 %
*Paracoccusyeei*	1	0.2 %
*Rhizobium radiobacter*	1	0.2 %

## Discussion

It is clear from the current research that hospital mattresses become significantly contaminated during use, even though they are covered with bed linens. These mattresses remain contaminated with bacteria, even after terminal cleaning with quaternary ammonia compound. The use of a launderable mattress cover helps prevent contamination of the hospital mattress and provides a less contaminated environment for patients.

Bacterial contamination in the hospital environment is a risk factor for HAIs [32]. Several authors have demonstrated that current cleaning protocols do not adequately decontaminate hospital rooms [6,8,33]. Hospital mattresses are often the most contaminated areas of hospital rooms [9,30]. Evaluating the hospital environment for contamination can be done by several published methods including bacterial sampling (using swabs, gauze, Dipslide, and RODAC) and measurement for organic matter (using adenosine triphosphate [ATP]) [7,8,12,18,26,28-30]. The current research utilized Petrifilm™ plates developed by 3 M Corporation for use in the food industry. Petrifilm™ plates do not require technical expertise to use or interpret. One other research team has used a different Petrifilm™ product, Staph Express Plates, to identify the presence of MRSA on egg-crate mattresses [31].

**Figure 2:**
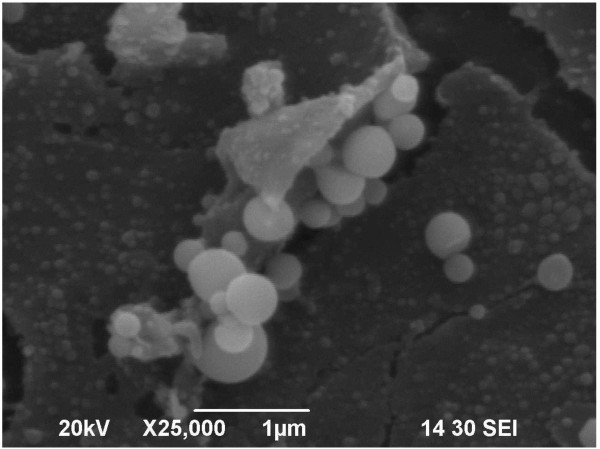
Scanning electron microscope picture of a polyurethane mattress surface showing cracking and bacterial contamination.

In the current study, terminal cleaning failed to eliminate bacteria from the surface of the mattress. Because of this contamination, mattresses that are damaged could allow for bacteria to contaminate the foam interior of the mattress, and this contamination has been linked to HAIs [13-19]. A scanning electron microscope picture of one of the mattresses utilized in the study shows the mattress cover to be both cracked and contaminated with bacteria (Figure 2). The research also demonstrated that the bed deck is more contaminated after terminal cleaning (Table 2). This may be a result of cross-contamination from the mattress surface.

A recent Field Safety Notice from Hill-Rom noted that beds “must be kept clean at all times in order to avoid a risk of infection. When cleaning, always check the cover for cuts, tears, cracks, pin holes or snags. Never use a mattress with a damaged cover—replace it before the inner core of the mattress becomes contaminated. If the internal foam of the mattress is soiled, you are advised to replace it as well.” [34]

The cleaner used in the current study was a quaternary ammonium compound (Quat), which is the most commonly used disinfectant in the United States [35]. The Environmental Protection Agency (EPA) has approved Quats for use on hard non-porous surfaces. The mattress surfaces are soft surfaces. The Quats have “a tendency to become attracted to and absorbed into fabrics.” [36] Quats are cationic (positively charged) and fabric is anionic (negatively charged), which can cause the chemicals to bind to the fabric and damage it.

In order to be classified as a disinfectant by the EPA, a product must “destroy or irreversibly inactivate infectious fungi and bacteria.” [37] In order to claim efficacy, the EPA requires that disinfectants kill at least 59 of 60 (98 %) samples placed on hard surfaces, and it must destroy more than 10^6^ organisms on each plate, after a 10 minute dwell time [38,39]. The EPA does not certify these chemicals for use on soft or porous surfaces, the type of surface generally found on hospital beds and furniture. In the current study, the disinfectant only decreased bacterial counts on the mattress surface by 84 %, and only 16 % of samples had no growth. This is concerning because, according to published reports, as few as 10 organisms can produce an infection, and bacteria can survive for months on dry surfaces [40-44].

The launderable mattress cover tested in the current study can be cleaned using heat and chlorine without damaging the cover. The manufacturer states that the cover could, if needed, be sterilized. The launderable covers were much cleaner than the surface of the terminally cleaned mattress. The cover also prevented the permanent mattress surface from contamination during use, which could have prevent transmission of bacteria to the next patient. Several studies have demonstrated the significantly increased risk of the subsequent patient contracting diseases when the previous patient had an infection with MRSA, VRE, or *C. difficile*[10-12].

The present research had limitations: (a) Only a small area of each mattress, bed deck or launderable cover was sampled. (b) The environmental services staff was aware of the purpose of the study, and they may have performed more effective cleaning during the study.

Most mattresses that have undergone terminal cleaning are still contaminated with bacteria. The newly-developed launderable cover provides a less contaminated surface and protects the mattress from contamination by the patient.

## Competing interest

Funding for the research was received by Xavier University from a private foundation on which an investor in Trinity Guardion, maker of a launderable mattress cover, serves.

## Authors’ contributions

EAH participated in the planning of the research, management of the project, data analysis, and manuscript preparation. SDA participated in the planning of the research, directed the laboratory analysis of microbiological growth, and participated in manuscript preparation. LDG participated in planning the research and manuscript preparation. CK participated in the planning of the research, performed the laboratory analysis, and participated in the manuscript preparation. All authors read and approved the final manuscript.
